# Correlation between syndecan-1 level and PELOD-2 score and mortality in pediatric sepsis

**DOI:** 10.5935/0103-507X.20210083

**Published:** 2021

**Authors:** Antonius Hocky Pudjiadi, Fatimah Saidah, Fatima Safira Alatas

**Affiliations:** 1 Department of Child Health, Cipto Mangunkusumo Hospital, Faculty of Medicine, Universitas Indonesia- Jakarta, Indonesia.

**Keywords:** Glycocalyx, Mortality, Organ dysfunction scores, Sepsis, Syndecan-1, Child

## Abstract

**Objective:**

To analyze the correlation between glycocalyx disruption measured via the serum syndecan-1 level and organ dysfunctions assessed by the PELOD-2 score and to evaluate its association with mortality in pediatric sepsis.

**Methods:**

We performed a prospective observational study in a tertiary public hospital. Sixty-eight pediatric patients diagnosed with sepsis according to International Pediatric Sepsis Consensus Conference criteria were consecutively recruited. We performed measurements of day 1 and day 5 serum syndecan-1 levels and PELOD-2 score components. Patients were followed up to 28 days following sepsis diagnosis.

**Results:**

Overall, the syndecan-1 level was increased in all subjects, with a significantly higher level among septic shock patients (p = 0.01). The day 1 syndecan-1 level was positively correlated with the day 1 PELOD-2 score with a correlation coefficient of 0.35 (p = 0.003). Changes in syndecan-1 were positively correlated with changes in the PELOD-2 score, with a correlation coefficient of 0.499 (p < 0.001) during the first five days. Using the cutoff point of day 1 syndecan-1 ≥ 430ng/mL, organ dysfunction (PELOD-2 score of ≥ 8) could be predicted with an AUC of 74.3%, sensitivity of 78.6%, and specificity of 68.5% (p = 0.001).

**Conclusion:**

The day 1 syndecan-1 level was correlated with the day 1 PELOD-2 score but not 28-day mortality. Organ dysfunction (PELOD-2 ≥ 8) could be predicted by the syndecan-1 level in the first 24 hours of sepsis, suggesting its significant pathophysiological involvement in sepsis-associated organ dysfunction.

## INTRODUCTION

Pediatric sepsis has an estimated annual global incidence of 1.2 million cases,^(^1^)^ accounting for 4% of hospitalizations and 8% of pediatric intensive care unit (PICU) admissions worldwide.^(^2^-^6^)^ Pediatric sepsis accounts for approximately 25% of hospital mortality.^(^5^)^ Multiple organ dysfunction (MODS) and septic shock are the major causes of mortality in sepsis.^(^7^-^9^)^

The main pathophysiological events leading to septic shock and MODS in sepsis are closely related to vascular endothelial dysfunction. The endothelial glycocalyx layer lines the vascular luminal side and plays a major role in maintaining vascular homeostasis, such as regulating endothelial permeability and leukocyte migration and inhibiting intravascular coagulation.^(^10^-^12^)^ Disruption of the glycocalyx layer on the vascular endothelium has been described as one of the main pathophysiological events in the development of shock and MODS via increased capillary leakage and microthrombus formation, eventually causing tissue hypoperfusion.^(^13^-^15^)^

Previous studies on rats have demonstrated degradation of the glycocalyx upon lipopolysaccharide-induced sepsis.^(^14^)^ The endothelial glycocalyx layer mainly comprises glycosaminoglycans, proteoglycans, membrane glycoproteins and plasma proteins. Transmembrane anchor proteins, syndecans, are the principal proteoglycans that maintain glycocalyx integrity,^(^16^)^ and their levels have been used to measure glycocalyx degradation. Previous clinical studies have demonstrated that a high serum syndecan-1 level is associated with capillary leakage and elevated inflammatory markers and correlated with the severity of sepsis.^(^9^,^15^,^17^)^

Endothelial glycocalyx disruption subsequently leads to increased vascular permeability, contributing to organ failure and mortality, and this has been described in many sepsis studies in adults.^(^10^,^11^,^18^)^ However, to date, the relationship between glycocalyx disruption and morbidity and mortality in pediatric sepsis remains unexplored. Evidence of glycocalyx involvement in pediatric sepsis could be a lead to improved diagnostic, prognostic, or therapeutic aspects to improve the outcome of sepsis in children.

Therefore, we aimed to investigate the role of glycocalyx degradation by measuring serum syndecan-1 levels in pediatric sepsis and its association with sepsis severity. We also investigated the prognostic value of the syndecan-1 level in terms of organ dysfunction measured by the Pediatric Logistic Organ Dysfunction-2 score (PELOD-2 score) and 28-day mortality.

## METHODS

We performed a prospective observational study in the PICU, emergency unit, and pediatric ward in Cipto Mangunkusumo Hospital, Jakarta, from March 2019 to March 2020. The study was reviewed by the Ethics Committee of the Faculty of Medicine, Universitas Indonesia, with approval number 0066/UN2. F1/ETIK/2019, January 2019.

A total of 68 pediatric patients were enrolled with prior parental consent obtained. The inclusion criteria were patients aged 1 month to 18 years diagnosed with sepsis. Sepsis was diagnosed according to the 2005 International Pediatric Sepsis Consensus Conference,^(^19^)^ and a procalcitonin level of > 2ng/mL was used as a marker of sepsis.^(^20^,^21^)^ Severe sepsis was defined as sepsis with the presence of at least one organ dysfunction based on the modified organ failure index developed by Doughty et al.^(^22^)^ Septic shock was defined as sepsis with cardiovascular dysfunction. The sepsis category for each subject was determined by at least two attending physicians outside the research team using the aforementioned criteria.

Postoperative cases; patients with autoimmune disease, malignancy, chronic kidney disease, or cyanotic heart disease; and patients transferred from the PICU of another hospital were excluded. Serum syndecan-1 levels and PELOD-2 scores were evaluated on day 1 and day 5 following the diagnosis of sepsis. Enrolled patients were followed up to 28 days for survival assessment. Nutritional status was assessed using actual body weight/ideal body weight for actual height and categorized under the Z score. The site of infection was determined by clinical and laboratory or radiological parameters supporting the diagnosis of a particular infection. Significant organ dysfunction was defined as a PELOD-2 score ≥ 8 as previously reported by Schlapbach et al.^(^23^)^

Blood samples were collected via vein puncture within 24 hours and subsequently within 5 x 24 hours following sepsis diagnosis. Syndecan-1 was centrifuged at 1000g for 10 minutes, and serum was transferred to Eppendorf tubes and stored at-80°C until analysis at the end of the study. Samples were tested in duplicate. Samples above the highest detection range of the kit were diluted and reran as required. Syndecan-1 was quantified via ELISA using Syndecan-1 (CD138) Human ELISA (Catalog number RGP009R, BioVendor, Brno, Czech Republic) performed at a laboratory partner, Prodia. The PELOD-2 score was assessed by at least two of the attending pediatric residents outside the research team to minimize observation bias. Laboratory examinations for PELOD-2 assessment, including lactate, serum creatinine, partial pressure of oxygen (PaO_2_) and partial pressure of carbon dioxide (PaCO_2_) as well as peripheral blood counts, were performed at the hospital clinical laboratory.

The minimum sample size for studying the correlation between the syndecan-1 level and PELOD-2 score was 42 patients, while that for the association of the syndecan-1 level and mortality was 40 patients. The sample size was calculated using a 5% significance, power of 80% and expected correlation coefficient of 0.4 based on a former study in adults.^(^24^)^ Missing data for any other reason apart from death under five days (prior to assessment for the day 5 syndecan-1 level and day 5 PELOD-2 score) were excluded from the analysis. Statistical analysis was performed using Statistical Package for the Social Sciences (SPSS) IBM, version 24.0 (IBM Corp, Armonk, USA). Normality testing was performed using the Kolmogorov-Smirnov test. Nonparametric data are presented as the median with a 25 - 75% interquartile range (IQR). A Spearman analysis was performed to evaluate the correlation of syndecan-1 and the PELOD-2 score. The statistical significance of differences for nonparametric quantitative data was determined using the Mann-Whitney U test. A receiver operating characteristics (ROC) curve was used to assess the efficacy of syndecan-1 in predicting the occurrence of significant organ dysfunction. The Youden index was used to determine the optimal cutoff point. A p value < 0.05 was considered significant.

## RESULTS

**Figure 1:**
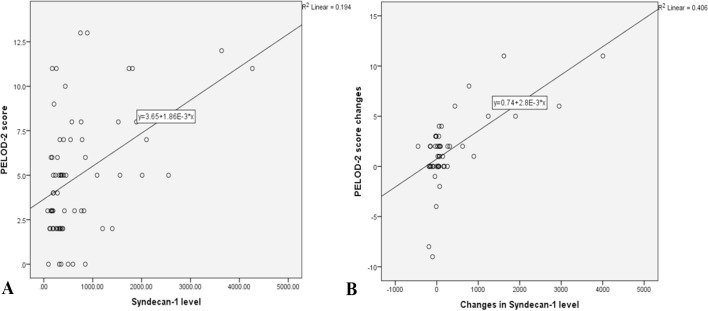
(A) Correlation between day 1 syndecan-1 level and day 1 PELOD-2 score (r = 0.35, p = 0.003). (B) Correlation between five-day changes in syndecan-1 levels and five-day changes in Pediatric Logistic Organ Dysfunction-2 scores (r = 0.499, p < 0.001).

**Figure 2:**
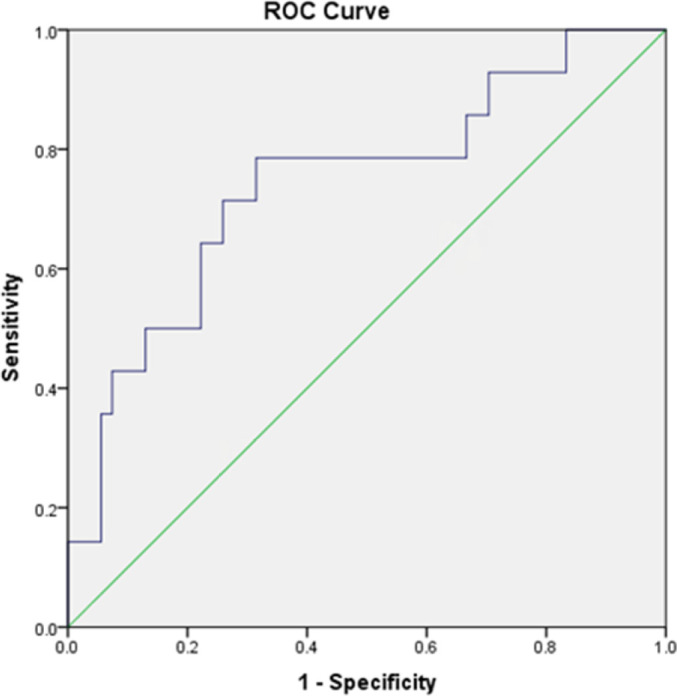
Receiver operating characteristic analysis of the day 1 syndecan-1 level for the prediction of significant organ dysfunction in the first 24 hours after the diagnosis of sepsis (area under the curve = 0.743).

We screened 85 patients during the study period, and 17 patients were excluded (nine did not have proven sepsis, and eight met the exclusion criteria upon work-up). Overall, sixty-eight patients were included in the analysis. Eighteen patients died before the fifth day, leaving only 50 patients available for the day 5 analysis. Survival data were obtained completely from the 68 patients. Most subjects were aged 1 month to 1 year old (52.9%) with the respiratory system as the primary infection site (66.2%). The median day 1 and day 5 syndecan-1 levels were 362.5ng/mL (IQR 196 - 811.5ng/mL) and 293ng/mL (IQR 175.5 - 542ng/mL) respectively. Two or more organ dysfunctions were observed for 65% of subjects on day 1 and for 28% of subjects on day 5. The 28-day mortality in this study population was 48.53%. Complete baseline characteristics are shown in table 1. There was an increased level of syndecan-1 in all subjects, with a significantly higher level found among patients with septic shock (p = 0.023, Table 2). There was a positive correlation between the day 1 syndecan-1 levels and day 1 PELOD-2 scores, with a correlation coefficient of 0.35 (p = 0.003, 95% confidence interval - 95%CI 0.12 - 0.54, Figure 1). Changes in syndecan-1 levels within 5 days were positively correlated with changes in PELOD-2 scores, with a correlation coefficient of 0.499 (p < 0.001, 95%CI 0.26 - 0.68, Figure 1). The day 5 syndecan-1 level was not significantly correlated with the day 5 PELOD-2 score (p = 0.6). There was no difference in day 1 syndecan-1 levels between subjects who survived and those who did not survive within the 28-day follow-up period (p = 0.23, Table 2). The day 1 syndecan-1 level could not predict 28-day mortality (p = 0.229, area under the curve - AUC 0.585, 95%CI (0.448 - 0.722)). There was no association between changes in syndecan-1 levels within 5 days and mortality at 28 days (p = 0.4). An ROC analysis was conducted to determine the best cutoff point of the day 1 syndecan-1 level to predict significant organ dysfunction. The cutoff point of the day 1 syndecan-1 level of ≥ 430ng/mL could predict significant organ dysfunction (PELOD-2 score of ≥ 8), with an AUC of 74.3%, sensitivity of 78.6%, specificity of 68.5%, positive predictive value of 39.3%, and negative predictive value of 92.5% (p = 0.001, 95%CI 0.58 - 0.89) (Figure 2).

PELOD-2 - Pediatric Logistic Organ Dysfunction-2.

**Table 1:** Baseline subject characteristics

Characteristics	Nonsurvivors (n = 33)	Survivors (n = 35)	p value
Sex male:female	19 (57.58):14 (42.42)	23 (65.71):12 (34.29)	0.660
Age category			0.116
1-month to 1-year	19 (57.58)	17 (48.57)	
> 1 - 5 years	8 (24.24)	12 (34.29)	
> 5 - 10 years	1 (3.03)	5 (14.29)	
> 10 - < 18 years	5 (15.15)	1 (2.86)	
Nutritional status			0.387
Obesity	1 (3.03)	0 (0.00)	
Overweight	0 (0.00)	2 (5.71)	
Normal	16 (48.48)	21 (60.00)	
Undernourished	8 (24.24)	5 (14.29)	
Severe malnutrition	8 (24.24)	7 (20.00)	
Fluid resuscitation	13 (39.39)	10 (28.57)	0.493
Mechanical ventilation	23 (69.70)	15 (42.86)	0.047*
Vasoactive use	15 (45.45)	11 (31.43)	0.347
Transfusion of blood products	25 (75.76)	20 (57.14)	0.172
Surgery	12 (36.36)	9 (25.71)	0.492
Infection site			0.741
Respiratory tract	20 (60.61)	25 (71.43)	
Gastrointestinal	7 (21.21)	7 (20.00)	
Central nervous system	3 (9.09)	2 (5.71)	
Urinary tract	2 (6.06)	1 (2.86)	
Skin	1 (3.03)	0 (0.00)	
Comorbidities (per organ system)†			0.437
None	5 (15.15)	9 (25.71)	
Cardiovascular	10 (30.30)	8 (22.86)	
Gastroenterology and hepatobiliary	10 (30.30)	12 (34.29)	
Malignancy	0 (0.00)	1 (2.86)	
Congenital anomalies/genetic diseases/syndrome	10 (30.30)	5 (14.29)	
Neurology and neuromuscular	8 (24.24)	6 (17.14)	
Respiratory	1 (3.03)	1 (2.86)	
Hematology and immune	0 (0.00)	1 (2.86)	
Urinary	0 (0.00)	1 (2.86)	
Nutrition and metabolic	9 (27.27)	7 (20.00)	
Serum lactate on day 1‡	2.65 (1.70 - 4.83)	1.80 (1.20 - 3.30)	0.053
Procalcitonin on day 1‡	12.16 (5.65 - 30.97)	16.08 (3.22 - 33.30)	0.300
C-reactive protein on day 1‡	73.50 (10.23 - 171.15)	41.10 (13.73 - 114.35)	0.183
PELOD-2 score on day 1‡	5 (2 - 6)	3 (2 - 5)	0.005*
PELOD-2 score on day 2‡	5 (2 - 7)	0 (0 - 3)	0.001*

PELOD-2 - Pediatric Logistic Organ Dysfunction-2. Categorical data were analyzed using a chi-squared test or Fisher’s exact test (if any cell count < 5). Results expressed as no (%) or median (interquartile range).

* p < 0.05; † One subject might have multiple conditions. ‡ p value for Mann-Whitney test.

**Table 2:** Comparison of day 1 syndecan-1 levels in different subject clinical categories

	n (%)	Syndecan-1 level (ng/mL)		p value
		Median	IQR	
Severe sepsis				0.193
Yes	51 (75)	391	208 - 1,089	
No	17 (25)	342	192 - 545	
Septic shock				0.023*
Yes	31 (45.6)	546	252 - 1,742	
No	37 (54.4)	342	191.5 - 545	
28-day mortality				0.23
Yes	33 (48.5)	404	278.5 - 849	
No	35 (51.5)	327	184 - 767	

IQR - interquartile range.

* p < 0.05.

## DISCUSSION

We measured day 1 and day 5 syndecan-1 levels as a marker for endothelial glycocalyx shedding and the PELOD-2 score as a tool to measure the severity of organ dysfunction in pediatric sepsis and found that the two were correlated. Elevated syndecan-1 was observed for all subjects, with significantly higher syndecan-1 in the septic shock clinical subgroup. Syndecan-1 was not associated with 28-day mortality but was able to predict the occurrence of severe organ dysfunction.

Our subjects had a median day 1 syndecan-1 level of 362.5ng/mL, with a range (min - max) of 76 - 4265ng/mL. Until now, there have been limited studies on syndecan-1 in the pediatric population. A study by Saragih et al. reported that the average syndecan-1 level in healthy children was 27.7ng/mL, with a level of > 41.42ng/mL (90^th^ percentile) indicating marked glycocalyx degradation.^(^25^)^ This suggests that syndecan-1 was increased for all pediatric sepsis subjects enrolled in this study. This is in line with several studies in adults, which reported increased levels of glycocalyx components such as syndecan-1 and hyaluronan during sepsis.^(^16^-^18^)^ Furthermore, we found significantly higher day 1 syndecan-1 levels in patients with septic shock (p = 0.02), similar to previous studies in adult sepsis.^(^16^,^17^,^26^)^ These results suggest that disruption of the glycocalyx contributes significantly to the pathophysiology of pediatric sepsis and is associated with the development of septic shock. Degradation of the glycocalyx exacerbates inflammation, increases vascular permeability, and disrupts the dilatation reflex of the vascular wall, resulting in septic shock.^(^11^)^

A positive correlation was found between the day 1 syndecan-1 levels and day 1 PELOD-2 scores. Previously, the correlation between glycocalyx disruption and organ dysfunction in sepsis was reported in adult patients.^(^16^,^24^)^ Anand et al. reported a positive correlation between the syndecan-1 levels in the first 24 hours of sepsis and organ dysfunction assessed with the Sequential Organ Failure Assessment (SOFA) score (r = 0.437).^(^24^)^ Kohler et al. also reported a correlation between syndecan-1 level and SOFA scores and Acute Physiology and Chronic Health Evaluation (APACHE) scores in the first 24 hours (r = 0.476 and 0.425, respectively).^(^18^)^ In this study, we also observed a significant moderate correlation between changes in syndecan-1 levels and changes in PELOD-2 scores within five days. These correlations between the syndecan-1 level and organ dysfunction scores support the role of glycocalyx degradation in the morbidity of organ failure in both adult and pediatric sepsis.

This study reports that the day 5 syndecan-1 level and day 5 PELOD-2 score were not significantly correlated (p = 0.6). This is contrary to Anand et al., whose study reported positive correlations between measured glycocalyx components and the SOFA score, which remained significant on days 1, 3, 5, and 7 following the diagnosis of sepsis.^(^24^)^ However, we observed a concurrent decreasing trend for both the syndecan-1 levels and PELOD-2 scores within five days in this study. The decrease in the PELOD-2 score occurred earlier than the decrease in syndecan-1 level. This may be confounded by the therapeutic effect of various interventions given to the patients, such as antibiotics, blood or albumin transfusion, which affect components of the PELOD-2 score and did not directly alleviate endothelial glycocalyx shedding.

The median day 1 syndecan-1 level was not significantly different between patients who survived and those who did not survive within the 28-day follow-up period (p = 0.23). Upon the subgroup analysis of those whose syndecan-1 increased, we also found no significant association with 28-day mortality (p = 0.4). Contrary to our finding, a previous study by Anand et al. reported significant associations between syndecan-1 levels and day 1, 3, 5, and 7 and 30-day mortality.^(^24^)^ This may be due to the confounding effect posed by the presence of underlying diseases or comorbidities, effects of various therapies, and biases of the cause of death other than sepsis that may affect these results. Our study was conducted in a tertiary referral hospital. There was high subject heterogenicity with clinical cases complicated by congenital diseases, chronic diseases, syndromes and nutritional problems. Such conditions might directly and/or indirectly affect disease progression and response to treatment, affecting overall mortality. Moreover, the effect of each comorbidity on endothelial glycocalyx shedding has never been studied. Therefore, we cannot draw conclusions on the association between day 1 syndecan-1 levels and 28-day mortality in this study.

We analyzed the use of day 1 syndecan-1 levels to predict significant organ dysfunction (PELOD-2 score > 8) using a ROC curve analysis. Using the optimal cutoff point for the day 1 syndecan-1 level of > 430ng/mL, significant organ dysfunction was predicted with a sensitivity of 78.6%, specificity of 68.5%, positive predictive value of 39.3%, and negative predictive value of 92.5% (p = 0.001, 95%CI 0.58 - 0.89). As sepsis is now defined as life-threatening organ dysfunction caused by a dysregulated host response to infection, organ dysfunction scores such as PELOD-2 in the pediatric population have become useful in the evaluation of pediatric sepsis. As the syndecan-1 level has a good predictive value for significant organ dysfunction, clinically, the use of syndecan-1 in pediatric patients may be useful to stratify pediatric sepsis patients based on severity and as a therapeutic guideline to avoid overtreatment.

To date, this is the first study of glycocalyx degradation in pediatric sepsis. However, there are some limitations to our study that we must address. The single-centered nature and subject heterogenicity in this study, as previously described, might have affected the clinical outcome of each subject. Furthermore, we only measured syndecan-1 on day 1 and day 5 after sepsis was diagnosed. This temporal mark was based on clinical diagnosis, which might have overlooked early or late presentation of actual sepsis progression. We recommend that future studies be conducted in multicenter settings with larger sample sizes. We also recommend comparing the use of other glycocalyx shedding markers, such as hyaluronan and heparan sulfate, to compare each validity to predict organ dysfunction and/or mortality in pediatric sepsis. Finally, different kinetics of glycocalyx shedding in pediatric sepsis with underlying comorbidities, as well as the effect of different treatments on glycocalyx shedding, should be investigated.

## CONCLUSION

In conclusion, glycocalyx degradation was observed in all pediatric sepsis subjects in this study. There was a positive correlation between the syndecan-1 level and the PELOD-2 score in the first 24 hours following the diagnosis of sepsis. The changes in syndecan-1 levels within 5 days were positively correlated with the changes in PELOD-2 scores. Both the day 1 syndecan-1 levels and five-day changes in syndecan-1 levels were not associated with 28-day mortality. We demonstrate that glycocalyx degradation plays a role to some extent in the pathophysiology of organ dysfunction in pediatric sepsis.

### Data availability statement

The data that support the findings of this study are available from the corresponding author, A. H. Pudjiadi, upon reasonable request.
